# Supplementary material for: Plasticity in structure and assembly of SARS-CoV-2 nucleocapsid protein
Source: bioRxiv. 2022 Feb 9:2022.02.08.479556. Preprint. [Version 1] doi: 10.1101/2022.02.08.479556 (PMC8845419; doi:10.1101/2022.02.08.479556)
Supplement: 1 [file NIHPP2022.02.08.479556V1-supplement-1.pdf]

bioRxiv preprint doi: https://doi.org/10.1101/2022.02.08.479556; this version posted February 9, 2022. The copyright holder for this preprint (which was not certified by peer review) is the author/funder. This article is a US Government work. It is not subject to copyright under 17 USC 105 and is also made available for use under a CC0 license.

Supplementary Information for

Plasticity in structure and assembly of SARS-CoV-2 nucleocapsid protein

Huaying Zhao[1], Ai Nguyen[1], Di Wu[2], Yan Li[3], Sergio A. Hassan[4], Jiji Chen[5], Hari Shroff[5,6], Grzegorz Piszczek[2], Peter Schuck[1]*

[1] Laboratory of Dynamics of Macromolecular Assembly, National Institute of Biomedical Imaging and Bioengineering, National Institutes of Health, Bethesda, MD 20892, USA
[2] Biophysics Core Facility, National Heart, Lung, and Blood Institute, National Institutes of Health, Bethesda, MD 20892, USA
[3] Proteomics Core Facility, National Institute of Neurological Disorders and Stroke, National Institutes of Health, Bethesda, MD 20892, USA
[4] Bioinformatics and Computational Biosciences Branch, National Institute of Allergy and Infectious Diseases, National Institutes of Health, Bethesda, MD 20892, USA
[5] Advanced Imaging and Microscopy Resource, National Institute of Biomedical Imaging and Bioengineering, National Institutes of Health, Bethesda, MD 20892, USA
[6] Laboratory of High Resolution Optical Imaging, National Institute of Biomedical Imaging and Bioengineering, National Institutes of Health, Bethesda, MD 20892, USA

*Correspondence: schuckp@mail.nih.gov

Supplementary Information contains

Tables S1 and S2

Figures S1 to S10

bioRxiv preprint doi: https://doi.org/10.1101/2022.02.08.479556; this version posted February 9, 2022. The copyright holder for this preprint (which was not certified by peer review) is the author/funder. This article is a US Government work. It is not subject to copyright under 17 USC 105 and is also made available for use under a CC0 license.

Table S1. Scope and depth of mutations in SARS-CoV-2 proteins

Protein	% of positions with mutations	average # of substitutions
E	77.3	2.48
M	71.6	2.28
N	86.4	3.49
S	79.3	2.87
ORF1a	86.9	2.67
ORF1b	75.6	2.33
ORF3a	97.8	4.02
ORF6	100	3.60
ORF7a	100	4.43
ORF7b	100	3.66
ORF8	98.4	3.74

Sequence data from GISAID, pre-processed and downloaded from Nextstrain.org on November 29th 2021, was parsed for the occurrence of mutations at each protein. Mutations were counted that appeared above a threshold of 10 times. At each position, the number of distinct substitutions was recorded and averaged over all positions that show any substitutions.

bioRxiv preprint doi: https://doi.org/10.1101/2022.02.08.479556; this version posted February 9, 2022. The copyright holder for this preprint (which was not certified by peer review) is the author/funder. This article is a US Government work. It is not subject to copyright under 17 USC 105 and is also made available for use under a CC0 license.

Table S2. Serine mutations in the SR-rich sequence

sequence	# of serine residues	# of sequences	% of sequences
no change (ancestral)	14	21,388	85.7% of all
any change		3,569	14.3% of all
≥1 S deletion	≤13	3027	12.1% of all
2 S deletions	12	69	**0.3% of all**<br>**2.3% of ≥1 deletion**
> 2 S deletions	<12	0	0%
≥1 new S position	≥15	603	2.4% of all
1 S deletion + ≥1 new S	≥14	61	**0.24% of all**<br>**2.0% of ≥1 deletion**

The number of serines occurring at positions 176-206 is counted in each of the 24,957 unique N sequences. In the ancestral sequence the number is 14. We count as a deletion of serine the mutation of a serine at specific position to any other amino acid. Of all sequences, 14.3% have at least one serine deleted, but of these only 2.28% have a second deletion. Thus, probabilities of deletion of serine are significantly lower for sequences with existing deletions (χ^2 = 315 (f=1), P < 1e-8), showing anti-correlation of serine mutations. *Vice versa*, insertion of a serine at a new position occurs only 2.42% of the time overall.

bioRxiv preprint doi: https://doi.org/10.1101/2022.02.08.479556; this version posted February 9, 2022. The copyright holder for this preprint (which was not certified by peer review) is the author/funder. This article is a US Government work. It is not subject to copyright under 17 USC 105 and is also made available for use under a CC0 license.

A

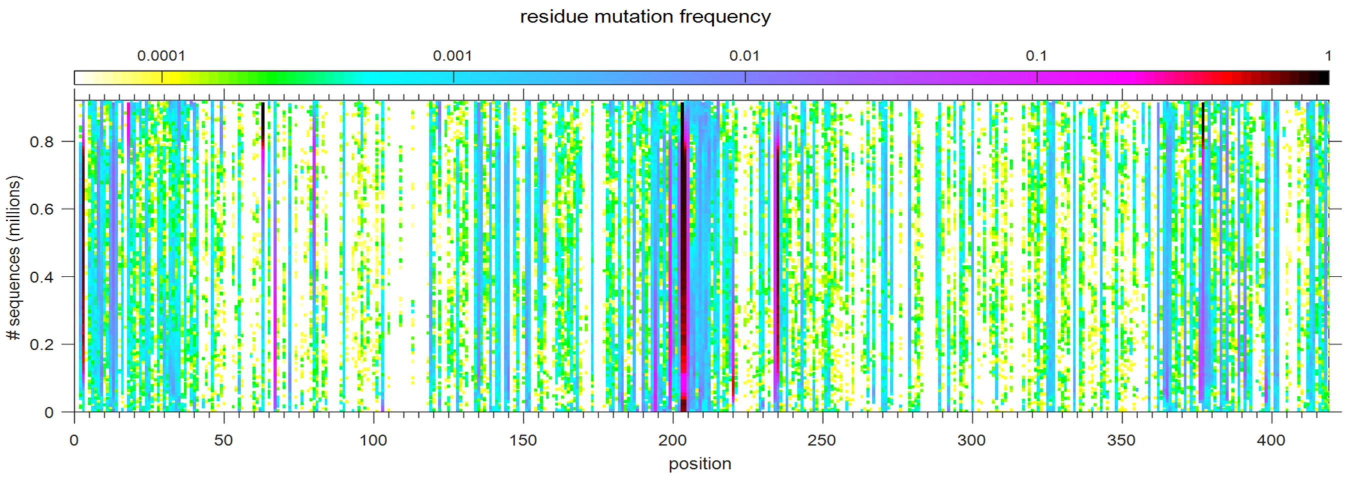

B

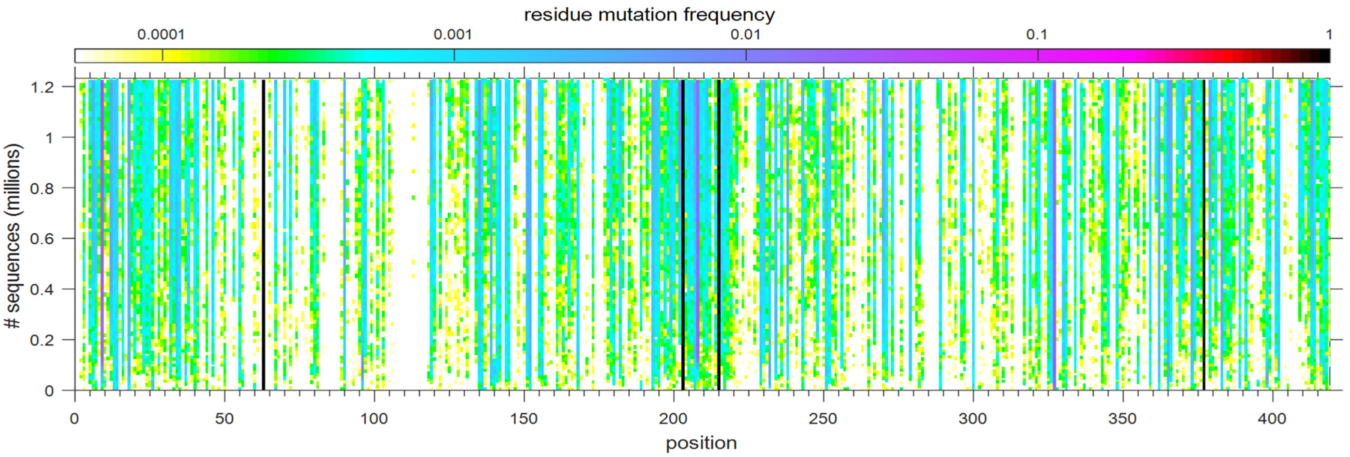

Figure S1. Temporal rate of mutations of variants. In an analogous presentation to **Fig. 3e**, shown is the frequency of mutations *vs.* total sequence number, subdivided for variants preceding Delta 21J (**A**) and Delta 21J (**B**). For each position, the daily number of any mutations relative to the number of new sequences, convoluted across 10^4 sequences, is plotted against the total accumulated sequences (as scale of time) and color-coded according to the relative rate of observing a mutation. The initial spread in the mutational landscape in shown **Fig. S2**.

bioRxiv preprint doi: https://doi.org/10.1101/2022.02.08.479556; this version posted February 9, 2022. The copyright holder for this preprint (which was not certified by peer review) is the author/funder. This article is a US Government work. It is not subject to copyright under 17 USC 105 and is also made available for use under a CC0 license.

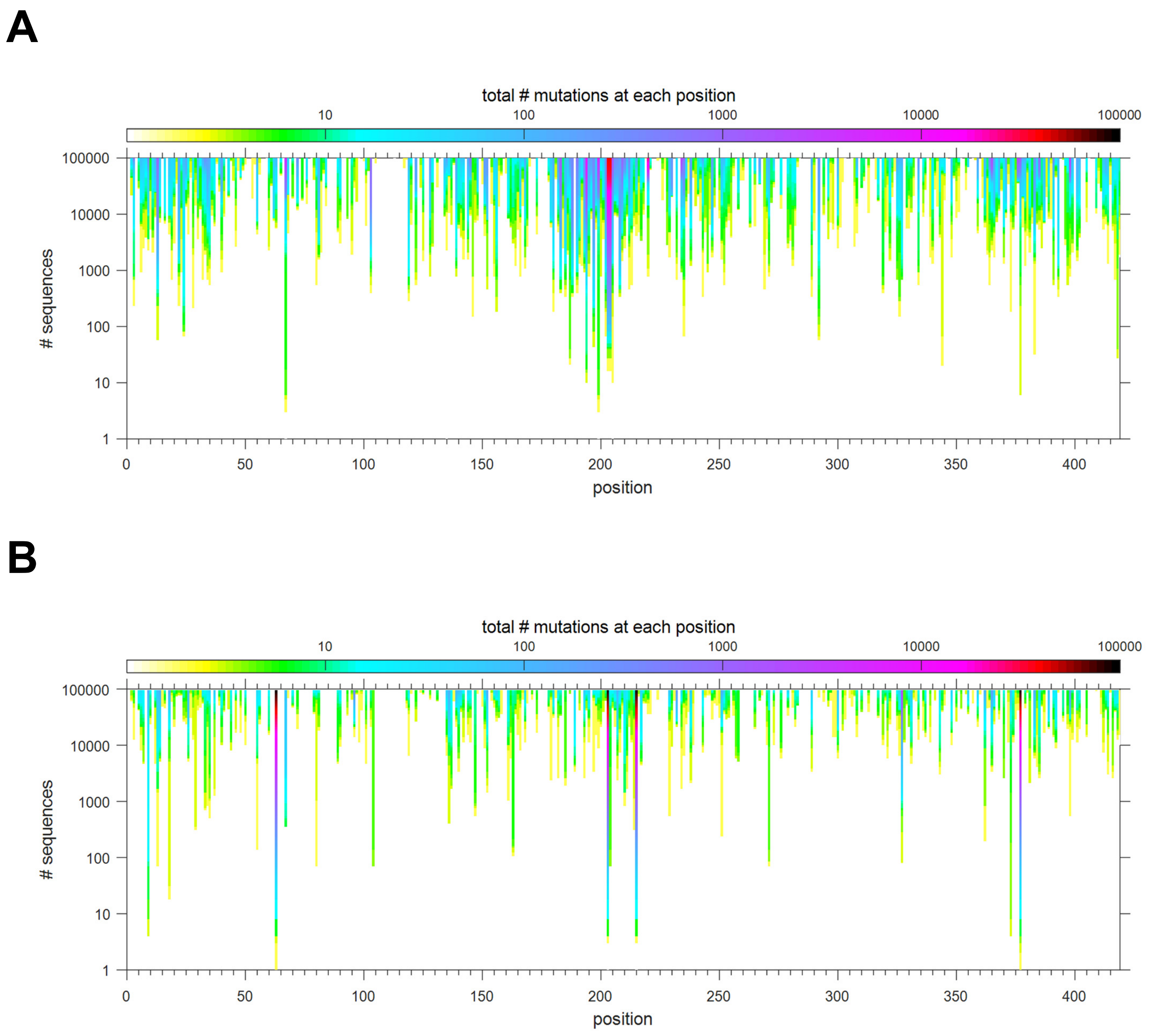

Figure S2. Spread of observed instances of mutations across sequence space. Shown is the accumulation of observed instances of mutations in each position, as a function of total number of sequenced genomes, for the first sequences of SARS-CoV-2 (**A**), and for Delta 21J (**B**). Initially only the most frequent mutations have been recorded, and more rare mutations are observed after many genomes have been sequenced.

bioRxiv preprint doi: https://doi.org/10.1101/2022.02.08.479556; this version posted February 9, 2022. The copyright holder for this preprint (which was not certified by peer review) is the author/funder. This article is a US Government work. It is not subject to copyright under 17 USC 105 and is also made available for use under a CC0 license.

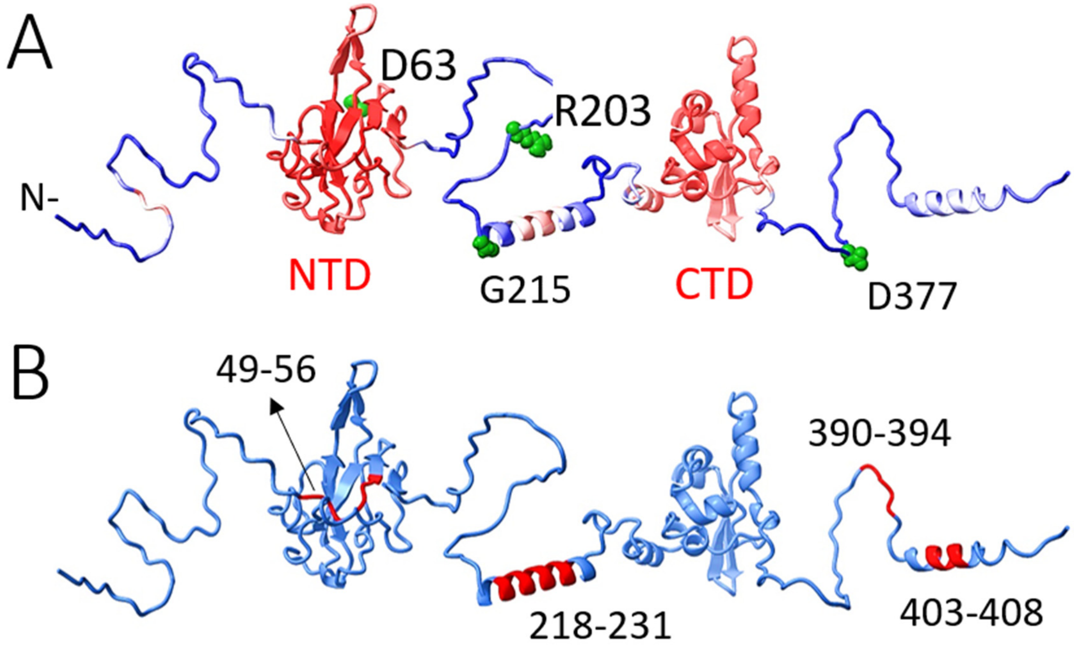

Figure S3. Three-dimensional model of full-length N-protein. (**A**) AlphaFold2 model of the N-protein (ancestral sequence) color-coded according to the prediction reliability (red: highest confidence; blue: lowest). While CTD and NTD are mostly red, the disordered regions (N-arm, linker, and C-arm) are mostly blue but show two short segments with helical content: G215-S235 and L400-S410. Similar helices in both positions are produced by independent calculations in structure prediction servers I-TASSER and Phyre2, albeit with low confidence, suggesting an intrinsic propensity for these short sequences to form helices within the highly disordered segment. Critical residues observed in the Delta variant are highlighted in green. The backbone of the disordered segments was rearranged relative to the folded domains for visualization purpose. (**B**) AF2 model showing in red the four regions most protected from mutations (yellow patches in Figure 3).

bioRxiv preprint doi: https://doi.org/10.1101/2022.02.08.479556; this version posted February 9, 2022. The copyright holder for this preprint (which was not certified by peer review) is the author/funder. This article is a US Government work. It is not subject to copyright under 17 USC 105 and is also made available for use under a CC0 license.

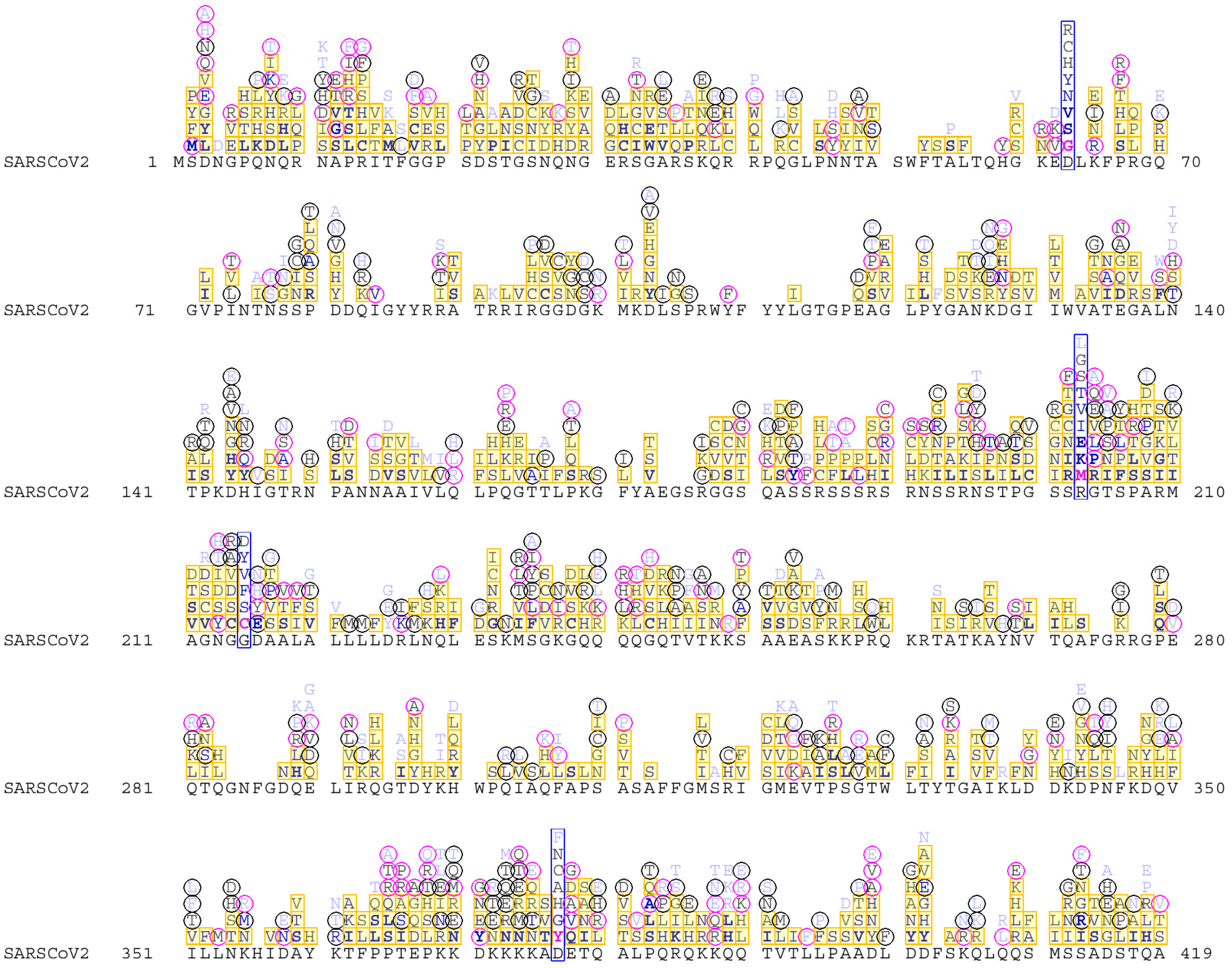

Figure S4. Amino acid substitutions in the mutational landscape of 21J Delta SARS-CoV-2 and non-21J Delta variants. The ancestral reference sequence Wuhan-Hu-1 is depicted in black letters, above which the range of observed mutations is presented, as in Extended Data Fig. 1 with different weights for different number of observations 10–20 (light blue), 20–100 (gray), 100-1,000 (blue), and > 1,000 times (bold blue). Mutations that occur in both 21J and non-21J sequences are highlighted in yellow squares, those occurring only in sequences from the 21J clade are encircled in black, and those only observed in non-21J sequences are encircled in magenta. The blue bars highlight the position of the four mutations characteristic for 21J Delta sequences. Mutations that have no highlight indicate rare mutations that only jointly exceed the threshold of 10 reports, but not in either the 21J or non-21J group alone.

bioRxiv preprint doi: https://doi.org/10.1101/2022.02.08.479556; this version posted February 9, 2022. The copyright holder for this preprint (which was not certified by peer review) is the author/funder. This article is a US Government work. It is not subject to copyright under 17 USC 105 and is also made available for use under a CC0 license.

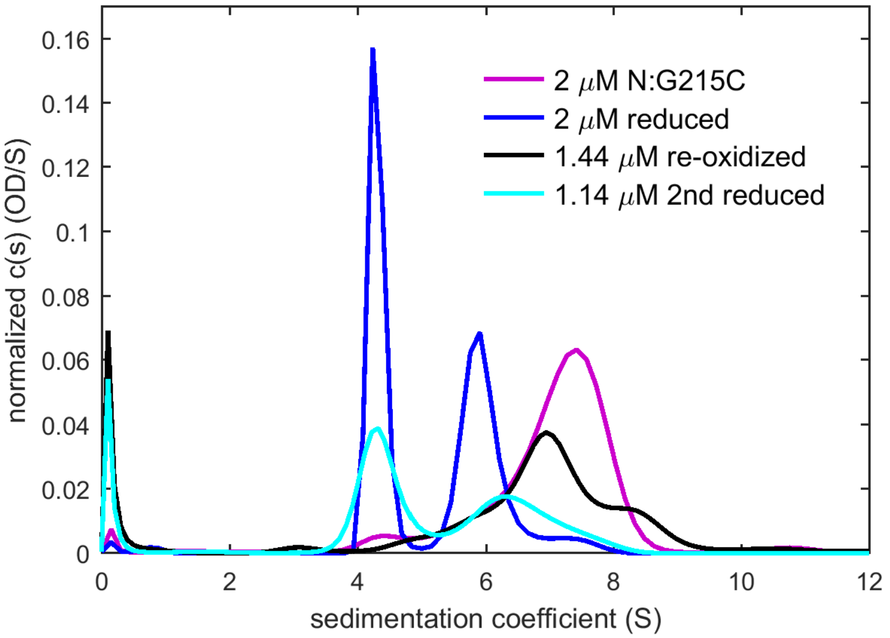

Figure S5. Oligomeric state of N:G215C after reversible reduction and re-oxidation. We asked whether the oligomeric distribution under non-reducing conditions might just be dynamically locked into the observed tetramer, and whether it is possible to alter it, if disulfide bonds are broken and then reformed. Initially, the original diluted protein stock is in a disulfide-linked tetrameric state (magenta), in a replicate experiment of **Fig. 5a**. Next, after dilution into 1 mM TCEP and incubation for 2 hours (blue) the majority of the protein is in a dimeric state (linked by CTD interactions) and exhibiting a reversible dimer-tetramer self-association equilibrium, replicating results of Fig. 3b. This is followed by removal of TCEP by overnight dialysis during which the protein is allowed to re-oxidize (black), which produces an assembled state, with an average s-value of 7.03 S virtually identical to the original state with 7.07 S. Based on hydrodynamic scaling laws, a hexamer would be expected to sediment at ~9.6 S, and a octamer at ~11.6 S. Importantly, the reformed disulfide bonds did not cause significant populations of oligomers larger than the tetramer. Finally, in order to demonstrate that these re-associated oligomers are disulfide-linked (as opposed to degraded misfolded oligomers), this sample was diluted again into 1 mM TCEP and incubated for 2 hours (cyan), reproducing the majority dimeric reduced protein state.

bioRxiv preprint doi: https://doi.org/10.1101/2022.02.08.479556; this version posted February 9, 2022. The copyright holder for this preprint (which was not certified by peer review) is the author/funder. This article is a US Government work. It is not subject to copyright under 17 USC 105 and is also made available for use under a CC0 license.

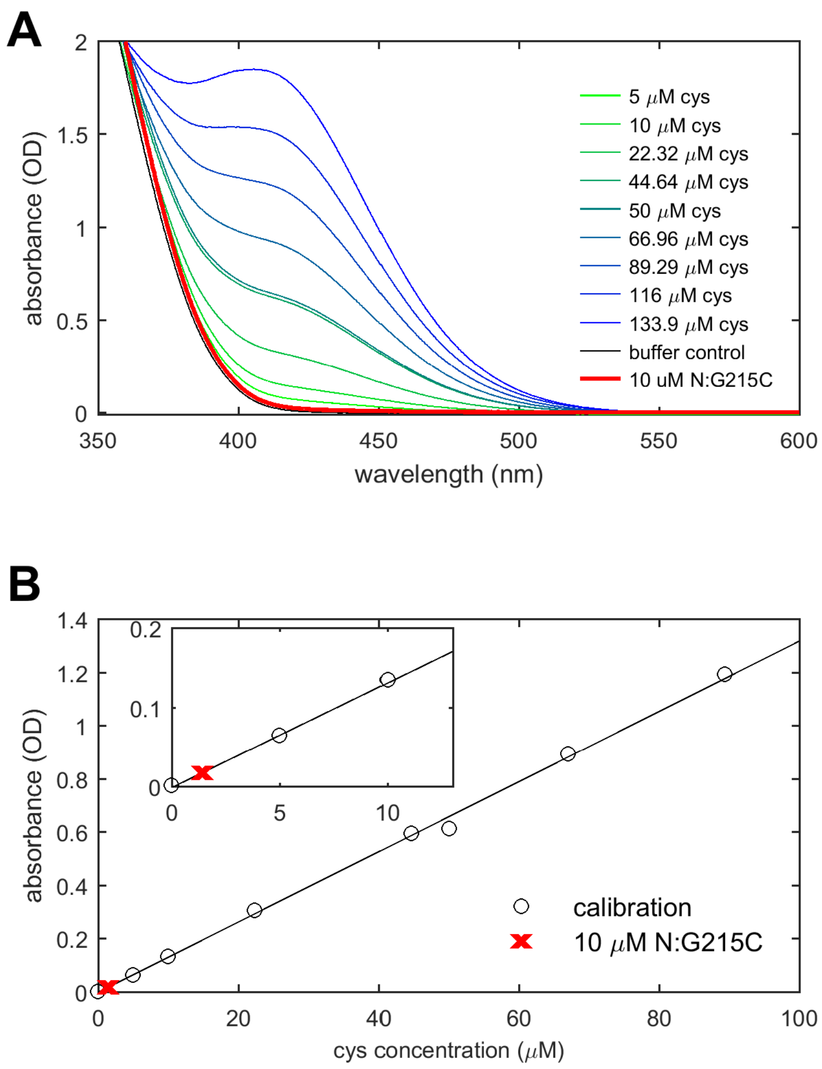

Figure S6. Free thiols assay determining the completion of disulfide bonds in N:G215C. (**A**) Absorption spectra of a concentration series of cysteine solutions after reaction with sulfhydryl reagent 5,5′-dithiobis (2-nitrobenzoic acid) (DTNB) (green to blue); and spectra of a buffer control and 10 μM N:G215C. (**B**) Conversion of spectral maximum at 412 nm into a standard curve (circles) and experimental value for 10 μM N:G215C, leading to an estimate of 1.4±0.1 μM free thiols in 10 μM N:G215C.

bioRxiv preprint doi: https://doi.org/10.1101/2022.02.08.479556; this version posted February 9, 2022. The copyright holder for this preprint (which was not certified by peer review) is the author/funder. This article is a US Government work. It is not subject to copyright under 17 USC 105 and is also made available for use under a CC0 license.

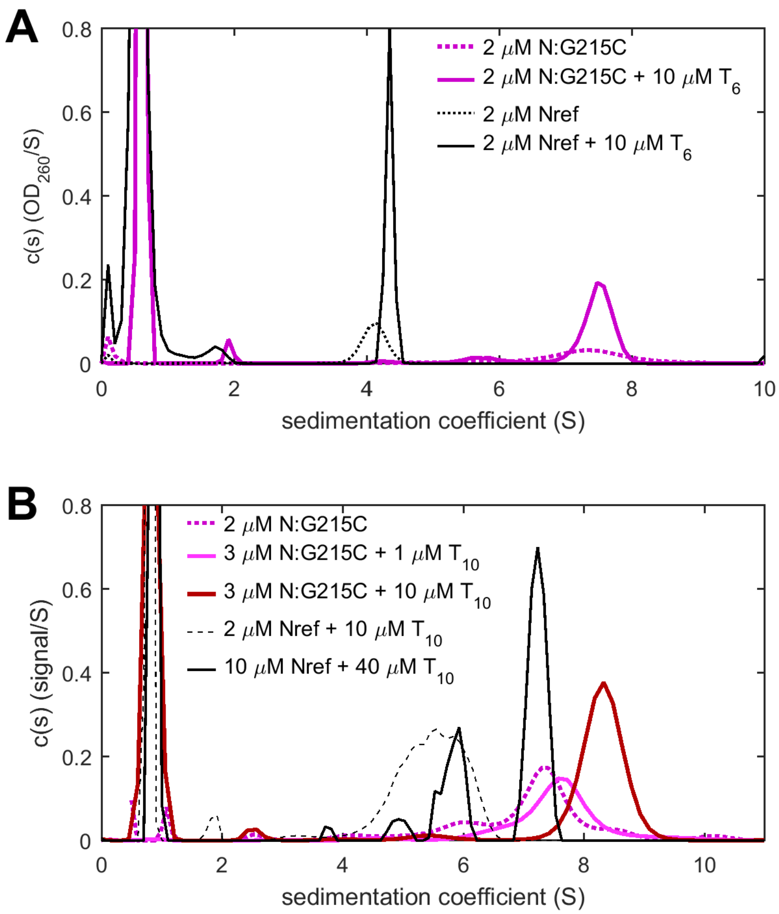

Figure S7. Nucleic acid binding to N:G215C oligomers. Binding is studied by SV using multiple detection systems simultaneously to measure NA binding while monitoring protein oligomeric states. (**A**) For reference, the hexa-oligonucleotide T_6 can ligand Nref dimers with a K_D of 0.6 μM at two sites (black solid and dotted lines for Nref with and without T_6) (Zhao et al., 2021a). The short oligonucleotide adds only little to the protein mass in the complex, and therefore does not significantly increase the sedimentation velocity. However, binding can be assessed from the increase of the 260 nm absorbance co-sedimenting with the complex. Therefore, sedimentation coefficient distributions c(s) were calculated based on sedimentation profiles acquired at 260 nm. For Nref the 260 nm absorbance increases 2.8fold due to bound T6 at the conditions shown (black solid line). For N:G215C, the increase in 260 nm absorbance is 2.3fold (magenta line), suggesting similar but slightly weaker binding. (**B**) Binding of the oligonucleotide T_{10} to Nref is close to stoichiometric at micromolar concentrations, and induces reversible N-protein dimer-dimer self-association (black lines). For Nref, the sedimentation coefficient distribution reflects time-average populations of dimer and tetramer species in rapid exchange, which exhibits a characteristic concentration dependence (Schuck and Zhao, 2017). For N:G215C, already at low concentrations of 2 μM N:G215C with 1 μM T_{10} (magenta) no free oligonucleotide is left, indicating high-affinity binding of T_{10} to N:G215C similar as to Nref. The oligomeric state of N:G215C liganded with T_{10} is tetrameric, similar to N:G215C in the absence of NA, with a slight shift consistent with the added mass from multiple T_{10} molecules. When applying a large molar excess of T_{10} (red), a larger shift is observed, consistent with added mass from up to eight copies of T_{10} expected to bind to the tetramer.

bioRxiv preprint doi: https://doi.org/10.1101/2022.02.08.479556; this version posted February 9, 2022. The copyright holder for this preprint (which was not certified by peer review) is the author/funder. This article is a US Government work. It is not subject to copyright under 17 USC 105 and is also made available for use under a CC0 license.

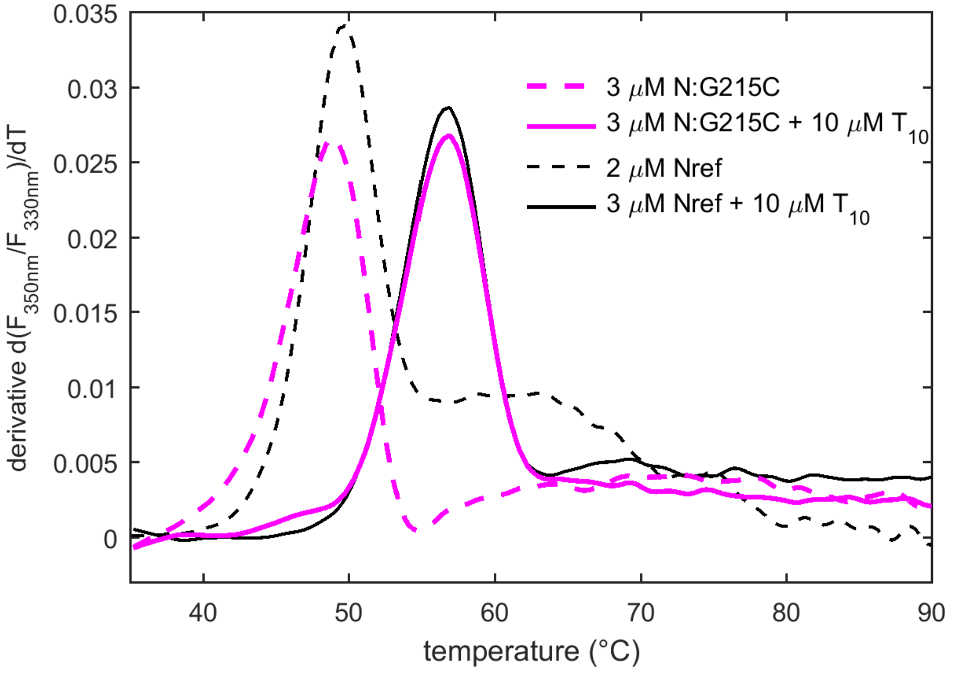

Figure S8. Thermal stability of N-protein and in complex with oligonucleotide T_{10}. Shown is the temperature-derivative of the intrinsic fluorescence ratio at 350 nm–330 nm (DSF) for Nref (black) and N:G215C (magenta) with and without molar excess of T_{10}. This data reflects on the thermal stability of the folded domains and their immediate vicinity, since tyrosine and tryptophan residues which are contributing to the recorded signal reside exclusively in the folded CTD and NTD domains. The shift to higher transition temperatures in the presence of T_{10} demonstrates higher thermodynamic stability of the complex.

bioRxiv preprint doi: https://doi.org/10.1101/2022.02.08.479556; this version posted February 9, 2022. The copyright holder for this preprint (which was not certified by peer review) is the author/funder. This article is a US Government work. It is not subject to copyright under 17 USC 105 and is also made available for use under a CC0 license.

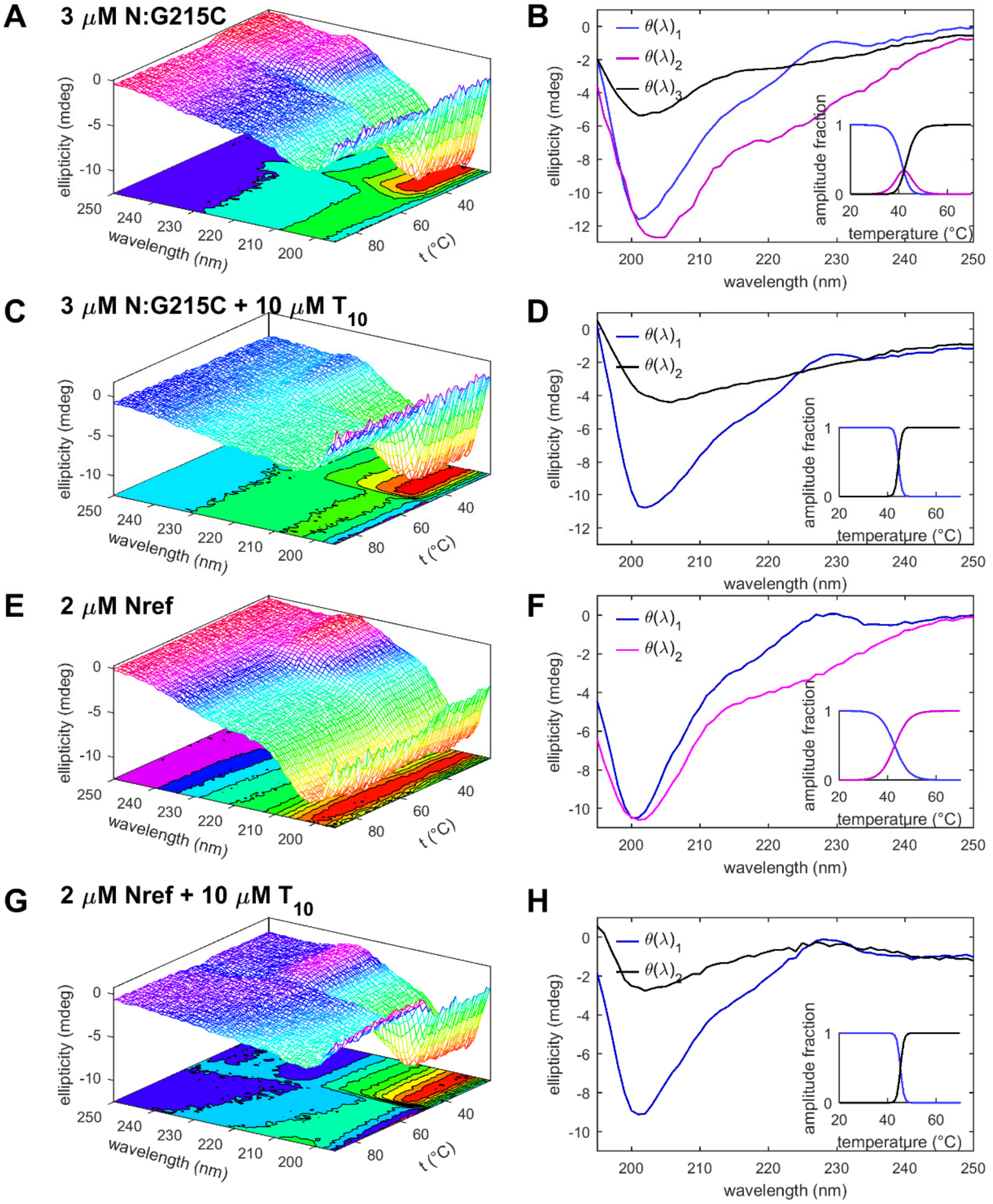

Figure S9. Temperature dependent secondary structure by CD. CD spectra were recorded as a function of temperature (left column) and decomposed into basis spectra (right column) and their relative contributions as a function of temperatures (insets). (**A**, **B**), CD data for 3 μM N:G215C, (**C**, **D**), 3 μM N:G215C with 10 μM oligonucleotide T_{10}, (**E**, **F**), 3 μM Nref, (**G**, **H**) 3 μM Nref with 10 μM T_{10}. N:G215C in working buffer (**A**, **B**) shows a transition at ~43°C from a largely disordered state (blue) to a state with increased negative 220-230 nm ellipticity characteristic for helical content (magenta). This mirrors the conformational changes for Nref (**E**, **F**), but for N:G215C it is followed quickly by a loss of signal (black) caused by depletion of material from the light path due to sedimentation of large particles formed. Similar depletion is observed for all CD data in the presence of T_{10}. Data for Nref are reproduced from Zhao et al. (Zhao et al., 2021a).

bioRxiv preprint doi: https://doi.org/10.1101/2022.02.08.479556; this version posted February 9, 2022. The copyright holder for this preprint (which was not certified by peer review) is the author/funder. This article is a US Government work. It is not subject to copyright under 17 USC 105 and is also made available for use under a CC0 license.

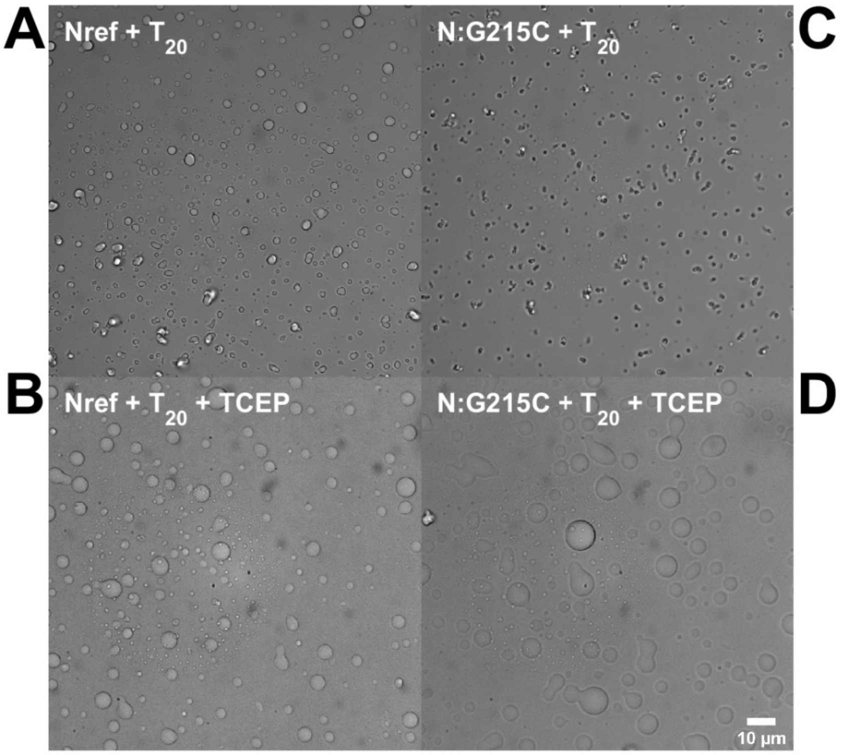

Figure S10. LLPS and particle formation of SARS-CoV-2. Shown are brightfield microscopy images of 5 µM Nref (left) and N:G215C (right) in presence of 10 µM oligonucleotide T_{20} in standard conditions (top) and reducing conditions of 1 mM TCEP (bottom). Under non-reducing conditions, for equivalent concentrations of N-protein and T_{20}, observed particles for N:G215C (**C**) are more fibrous than those for Nref (**A**). Such morphology can also be observed for Nref under slightly different conditions, and has been reported by others (Carlson et al., 2020). Addition of 1 mM TCEP as a reducing agent slightly accelerated droplet formation for Nref (**B**). Since Nref does not have any cysteines, this effect emphasizes the dependence of LLPS on protein solvation; for example, preferential exclusion of co-solutes from the vicinity of the protein will tend to stabilize the dense phase. For N:G215C the presence of 1 mM TCEP leads to reduction of disulfide bonds and altered quaternary structure. In the presence of T_{20} this promotes the formation of slightly larger droplets for reduced N:G215C (**D**) than for Nref (**B**).
